# Loading of Silver (I) Ion in L-Cysteine-Functionalized Silica Gel Material for Aquatic Purification

**DOI:** 10.3390/gels9110865

**Published:** 2023-10-30

**Authors:** Mohammed A. Al-Anber, Malak Al Ja’afreh, Idrees F. Al-Momani, Ahmed K. Hijazi, Dinara Sobola, Suresh Sagadevan, Salsabeel Al Bayaydah

**Affiliations:** 1 Laboratory of Inorganic Materials and Polymers, Department of Chemistry, Faculty of Sciences, Mutah University, P.O. Box 7, Al-Karak 61710, Jordan; 2 Department of Chemistry, Faculty of Sciences, Yarmouk University, Irbid 21163, Jordan; 3 Department of Chemistry, Faculty of Sciences and Arts, Jordan University of Science and Technology, Irbid 22110, Jordan; akhijazi@just.edu.jo; 4 Department of Physics, Faculty of Electrical Engineering and Communication, Brno University of Technology, Technická 2848/8, 61600 Brno, Czech Republic; 5 Institute of Physics of Materials, Czech Academy of Sciences, Žižkova 22, 61662 Brno, Czech Republic; 6 Nanotechnology & Catalysis Research Centre, University of Malaya, Kuala Lumpur 50603, Malaysia

**Keywords:** silver (I) Ion, L-cysteine, silica gel, Langmuir isotherm, thermodynamic parameters

## Abstract

The L-cysteine-functionalized silica (SG-Cys^−^Na^+^) matrix was effectively loaded with silver (I) ions using the batch sorption technique. Optimal Ag(I) loading into SG-Cys^−^Na^+^ reached 98% at *p*H_i_ = 6, 80 rpm, 1 mg L^−1^, and a temperature of 55 °C. The Langmuir isotherm was found to be suitable for Ag(I) binding onto SG-Cys^−^Na^+^ active sites, forming a homogeneous monolayer (*R*^2^ = 0.999), as confirmed by FTIR spectroscopy. XRD analysis indicated matrix stability and the absence of Ag_2_O and Ag(0) phases, observed from diffraction peaks. The pseudo-second-order model (*R*^2^ > 0.999) suggested chemisorption-controlled adsorption, involving chemical bonding between silver ions and SG-Cys^−^Na^+^ surface. Thermodynamic parameters were calculated, indicating higher initial concentrations leading to increased equilibrium constants, negative Δ*G* values, positive Δ*S* values, and negative Δ*H*. This study aimed to explore silver ion saturation on silica surfaces and the underlying association mechanisms. The capability to capture and load silver (I) ions onto functionalized silica gel materials holds promise for environmental and water purification applications.

## 1. Introduction

Functionalized silica gel matrices are silica gel structures that have been modified or functionalized with specific chemical groups or molecules. These modifications can impart new properties or enhance the existing properties of the silica gel matrix, expanding its range of applications to ion exchange matrices [1], chromatography matrices [2], affinity matrices [3], catalytic matrices [4], drug delivery matrices [5], and adsorbent matrices [6]. The choice of specific functionalization methods and chemical groups depends on the desired properties and applications of the silica gel matrix. The sorption of silver ions onto the functionalized silica gel involves the incorporation of silver ions into the modified silica gel matrix. Functionalized silica gel matrices, including silver ions, are useful in a variety of applications such as antibacterial agents [7], water purification [8], catalysis [9], gas sensors [10], and optical devices [11]. It is worth noting that the specific applications and performance of silica gel matrices functionalized with silver ions may vary depending on the synthesis method, silver ion loading, and other factors.

It is known in the literature; silver ions can be found in several materials such as polyurethanes modified by carbamide or amino groups [12], poly(aniline-co-5-sulfo-2-anisidine) nano sorbents [13], Na-exchanged clinoptilolite [14], coconut fiber [15], a copolymer of 1-vinyl-1,2,4-triazole with acrylonitrile [16], bentonite clays [17], and mesoporous silica with dendrimer amines [18], N-(2-(2-pyridyl)ethyl)chitosan against [19], sulfonated calix[4]arene [20], O-carboxymethyl chitosan beads grafted with thiourea-glutaraldehyde [21], thiourea-immobilized polystyrene nanoparticles [22], amino-carbamate moiety [23], thiourea-grafted polymeric resin [24], poly(hexamethylene guanidine) [25], dithiooxamidated polysiloxane [26], dimercaptotriazine functionalized silica [27], silica surface hydrosol [28], modified mesoporous silica microcubes [29], and amino-functionalized nanoporous silicas [30]. 

Silver ions are known for their antibacterial properties, and their release into the environment could adversely affect ecosystems. Silver ions can be discharged into water streams from various sources, primarily as a result of human activities and industrial processes. Some consumer products, such as antimicrobial textiles, personal care products, and water purification systems, contain silver-based materials. Furthermore, silver is present in various electronic devices. Improper disposal and recycling of electronic waste can result in the leaching of silver and other metals into the environment. The toxicity of silver ions in water can vary depending on several factors, including the concentration of silver ions present and the exposure duration. The lethal concentration of silver ions in aquatic environments falls within the range of 1 to 100 μg/L for sensitive species. In aquatic environments, high concentrations of silver ions can be toxic to aquatic organisms. Silver ions can disrupt cellular processes and impact the functioning of biological systems. The exact threshold concentration for toxicity can vary between different species. It’s important to note that regulations and guidelines exist in many countries to limit the concentration of silver ions in water to safe levels. These guidelines help prevent potential harm to human health and the environment. The acceptable concentration limits can depend on the intended use of water (e.g., drinking water, recreational water) and the specific regulations of the region. In general, while silver ions have beneficial antimicrobial properties, their toxicity in water underscores the need for careful management and control of their release into aquatic environments to ensure the safety of both ecosystems and human populations [31].

The extensive exploration of various solid materials for capturing and immobilizing silver ions in aqueous solutions has not adequately considered L-Cysteine-functionalized silica gel materials for aquatic purification. While alternative sorbents and ion-exchange materials have been studied for capturing silver ions, there’s a notable lack of comprehensive research into the potential benefits, efficiency, and unique properties of L-Cysteine-functionalized silica gel materials in this context. Addressing this research gap calls for an in-depth investigation into the feasibility and effectiveness of using L-Cysteine-functionalized silica gel as a solid material for capturing silver ions in aquatic environments. Such a study could examine ion exchange and/or sorption capacity, thermodynamics, kinetics, and the characterization of L-Cysteine-functionalized silica gel materials including silver ions. Additionally, comparing L-Cysteine-functionalized silica gel with existing sorbents mentioned in the literature would offer valuable insights into its advantages and limitations, aiding the development of more efficient and sustainable capturing silver ion methods for environmental remediation.

The ability to capture and load silver (I) ions onto a functionalized silica gel material could be valuable in environmental and water purification applications. Using functionalized silica gel to capture and remove silver ions could be an eco-friendly and efficient way to mitigate their harmful effects. Herein, we are interested in the utilization of l-cysteine-functionalized silica gel (SG-Cys^−^Na^+^) against full-loaded silver ions. The binding mechanism of silver ions into silica gel can lead to the formation of a silver-silica composite material. Understanding the binding mechanism of silver ions into the SG-Cys^−^Na^+^ surface allows for the design and development of functional materials with various applications. For example, the load of silver ions onto a functionalized silica gel material could enhance the antimicrobial properties and may be used to disinfect or treat water. The mechanism is affected by several parameters such as pH, initial concentrations, and temperature. Therefore, it is important to consider the parameters of the requirements to optimize the performance of SG-Cys^−^Na^+^ materials. The data from the different experiments can be examined using adsorption kinetics and isotherm models.

## 2. Experimental Section

Silver nitrate (AgNO_3_, 98%) and L-cysteine-functionalized silica gel (SG-Cys^−^Na^+^, ≥99.0%) were purchased from Sigma-Aldrich Chemie GmbH, Taufkirchen, Germany). Sodium hydroxide (NaOH, 99.9%) was purchased from Fluka Chemika Commercial Providers. Nitric acid 65.0% (HNO_3_) was purchased from ACS Chemicals (Ahmed Abad, India).

### 2.1. Instrumentation and Analysis

Atomic Absorption Spectrophotometer, (Varian Spectra AA 55, Perkin-Elmer, Waltham, MA, USA) was used to analyze the silver ion concentration in the aqueous solution. The pH of all solutions was recorded by pH meter (Orion 520). A temperature controller controlled the temperature (Gesellschaft Funn 1003, ±0.1 °C). An isothermal shaker was also used (Gesellschaft Fur 978). The analytical balance is used with ±0.0001 mg (Sartorius, CP324-S/management system certified according to ISO 9001). Attenuated total reflectance infrared spectroscopy (ATR-IR), (Alpha, Bruker, Mannheim, Germany) was used to analyze and study the changes in the functional groups. SEM images were taken on SEM model Quanta FEG 450, (Germany). Before analysis, the sample was deposited onto a double stick tape fixed on an aluminum sample holder and was then coated with gold in a sputter coater. Thermogravimetric analysis (TG), differential thermogravimetric (DTG), and differential thermal analysis (DTA) of SG-Cys^−^Na^+^ were recorded on a PCT-2A thermo-balance analyzer. The rate of heating is selected at 10 °C per minute and the sample is heated up to 1073 K under an N_2_ atmosphere. The known weight of the sample is about 2.5–5.0 mg. X-ray diffraction technique (XRD) was carried out using XRD Rigaku Ultima IV device by Sonneveld-Visser’s method with X-ray Cu/40 kV/40 mA, Goniometer Ultima IV (185 mm), and K -Beta filter.

### 2.2. Batch Sorption Experiments

The sorption performance of the SG-Cys^−^Na^+^ matrix toward silver (I) ions was tested using a batch system at specific temperatures and variable concentrations of silver (I) ions. The closed capture system containing 2 g L^−1^ (0.1 g/50 mL) of SG-Cys^−^Na^+^ was shaken vigorously (80 rpm) and controlled for up to 180 min. The pH of the solution was adjusted to 2, 3, 5, and 6 by using either 0.1 M NaOH or 0.1 M HNO_3_. The supernatant solution was then filtered. Atomic Absorption Spectrophotometer, (Varian Spectra AA 55, Perkin-Elmer, USA) was used to analyze the silver ion concentration in the aqueous solution. The obtained experimental results were used to study kinetic and isotherm models. was expressed as the amount of silver (I).

## 3. Result and Discussion

**Scheme 1:**
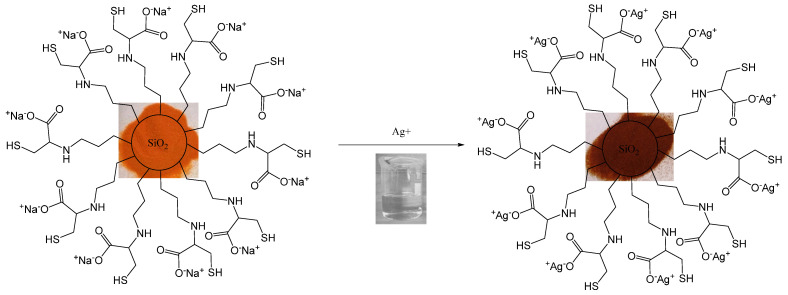
Capturing and Loading of Silver (I) Ion into L-Cysteine-Functionalized Silica Gel Material.

The binding mechanism of silver ions to SG-Cys^−^Na^+^ can lead to the formation of a silver-silica composite material. The binding of silver ions to SG-Cys^−^Na^+^ typically occurs through chemical interactions such as ion exchange and coordination bonding. The functional active sites in SG-Cys^−^Na^+^, such as carboxylate, amine, and thiol, make it an excellent candidate for immobilizing silver ions in the silica gel matrix. The chemical interaction of silver (I) ions with SG-Cys^−^Na^+^ can be attributed to several factors such as pH, temperature, contact time, and initial concentration of metal ions. However, electrostatic interactions, coordination bonding, and ion exchange can alter the behavior of these interactions. Silver (I) ions carry a positive charge; thus, they can interact with negatively charged functional groups through electrostatic attractions. These electrostatic interactions facilitated the capture of silver (I) ions onto the SG-Cys^−^Na^+^ material. SG-Cys^−^Na^+^ possesses ion-exchangeable sites on its surface. Since both ions have the same charge, the difference in charge density might not be substantial enough to be a significant factor in the ion exchange process. However, the larger ionic radius of Ag(I) (due to its higher effective nuclear charge) might affect its ability to replace Na^+^ in certain SG-Cys^−^Na^+^ active sites. In this case, silver (I) ions can replace sodium cations present on the SG-Cys^-^Na^+^ material through ion-exchange reactions (SG-Cys^−^Na^+^ (s) + Ag^+^ (aq) → SG-Cys^−^Ag^+^ (s) + Na^+^ (aq)), leading to their capture as shown in Scheme 1. 

### 3.1. ATR-FTIR

**Figure 1:**
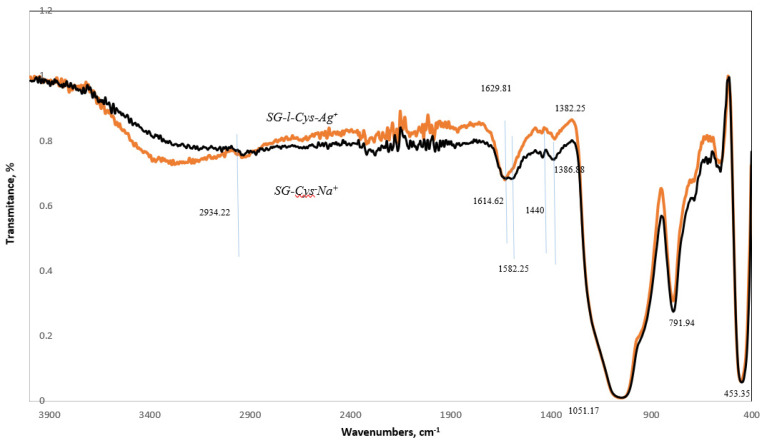
FTIR of the SG−Cys^−^Na^+^ and SG−Cys^−^Ag^+^.

Figure 1 shows two type bands I and II of the infrared (IR) features related to the SG-Cys^−^Na^+^. The Type-I band typically occurs at approximately 1614 cm^−1^ and corresponds to the stretching vibration of the carboxylate C=O and C-O bonds. Type II band appears at approximately 1582 cm^−1^ and arises from a combination of N-H bending and C-N stretching vibrations. In addition, the peaks at 2934 cm^−1^ correspond to the absorption bands of the CH2^−^ groups. The interaction between the Ag(I) ion and L-cysteine can give rise to characteristic peaks corresponding to SG-Cys^−^Ag^+^ bonds. The exact positions of the Type-I band shifted to a higher frequency (1630 cm^−1^), while no changes were observed for the other functional groups. These results demonstrate that Ag(I) can be successfully captured by SG-Cys^−^Na^+^ through the carboxylate sites. Generally, these peaks depend on specific silver ions and their coordination geometry. Finally, the silica gel matrix may also contribute to the infrared spectrum, typically showing the symmetric stretching vibration of Si-O-Si at 1051 cm^−1^. The bands at 792 and 453 cm^−1^ correspond to the asymmetric stretching and bending modes of Si-O. These findings are consistent with those previously reported [32]. 

### 3.2. Thermogravimetric Analysis

**Figure 2:**
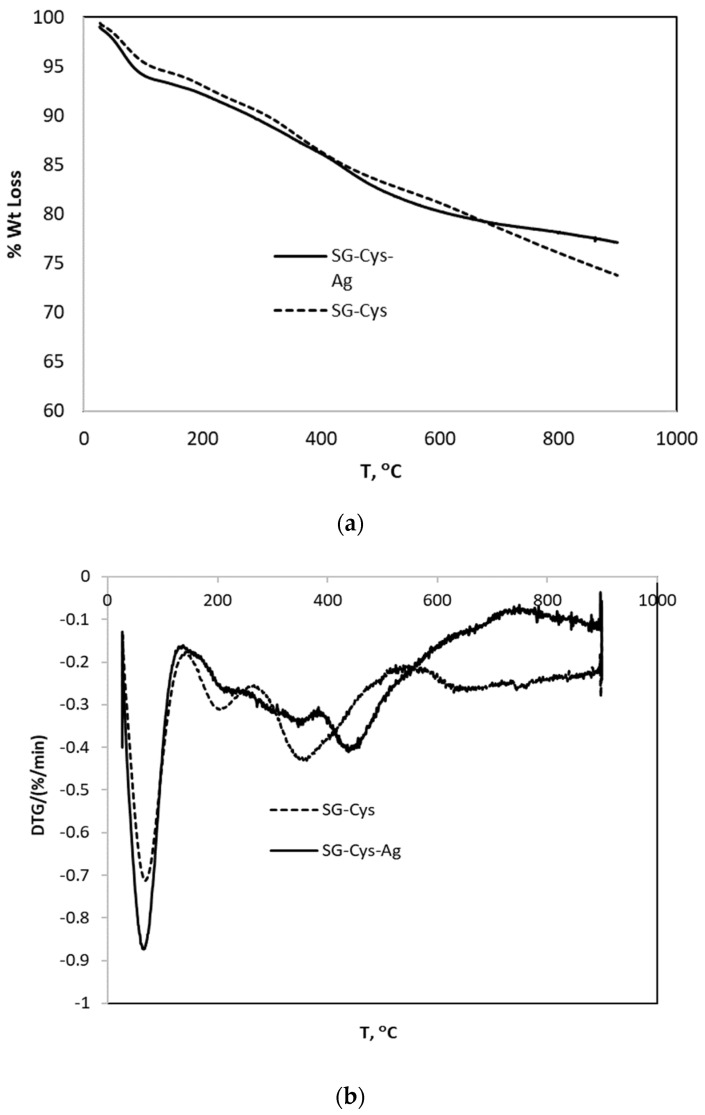
(**a**). Thermogravimetric Analysis of SG−Cys^−^Na^+^ and SG−Cys^−^Ag^+^. (**b**). Differential thermal analysis (DTA) for the SG−Cys^−^Na^+^ and SG−Cys^−^Ag^+^.

Thermogravimetric analysis (TGA) and differential thermal analysis (DTA) of SG-Cys^−^Na^+^ and SG-Cys^−^Ag^+^ are shown in Figure 2a and Figure 2b, respectively. The thermal characteristics and behaviors are shown in Figure 2a. In this case, the overall weight loss recorded was 21.5% of the weight taken. The observed weight reduction is comparable to that reported previously [16]. The TG curve indicates a constant and fast initial weight loss of 5.5% at temperatures less than 100 °C, which can be attributed to the elimination of physisorbed water or volatile chemicals adsorbed on the surface of SG-Cys^−^Na^+^ and SG-Cys^−^Ag^+^. The elimination of chemisorbed water may be responsible for the second gradual and delayed weight loss of 3.3% at 220 °C [17]. This stage is normally reached at a temperature of 360 °C. Weight loss is due to the breakdown of cysteine molecules and the production of volatile chemicals. The stability of the silica gel backbone can be seen as a plateau or steady weight loss at higher temperatures, with the remaining 78.5% up to 700 °C. DTA (Figure 2b) revealed that all weight losses were exothermic. This thermal behavior was observed for octakis(tetramethylammonium)-t8-silsesquioxane (octa-anion) in porous silica gel [33]. 

The thermal behavior of SG-Cys^−^Ag^+^ is shown in Figure 2a. The total weight loss recorded was 21.0% of the weight taken. The first steady and sharp initial weight loss of 6.57% at temperatures lower than 100 °C can be associated with the removal of physisorbed water or volatile compounds adsorbed on the surface of SG-Cys^−^Ag^+^. The second gradual weight loss of 2.45% at 224 °C may be associated with the removal of chemisorbed water. The third weight loss of 4.37% at 354 °C is related to the decomposition of cysteine, which does not bind to silver ion components. The fourth weight loss of 4.55% at 448 °C is related to the decomposition of cysteine binding with the silver ion components. The stability of the silica gel backbone can be observed by a plateau or gradual weight loss at higher temperatures, with the remaining 78.96% up to 700 °C. DTA (Figure 2b) shows that all weight losses are exothermic. 

As a comparison between TGA and DTA for both SG-Cys^−^Na^+^ and SG-Cys^−^Ag^+^, we found that silver ions enhanced the thermal properties of the cysteine organic components, increasing the thermal decomposition from 354 to 448 °C. Moreover, we may conclude that the percentage of reacted cysteine is 0.18%, in contrast to the non-reacted cysteine. The percentage of silver components that reacted with SG-Cys^−^Na^+^ was 3%.

### 3.3. Morphology of the Surface

**Figure 3:**
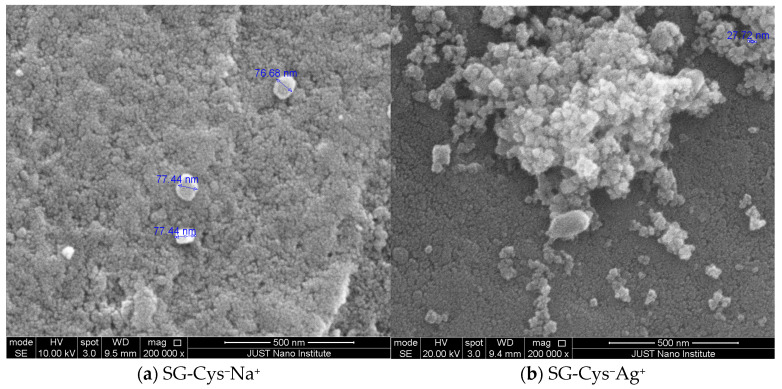
The SEM images of (**a**) SG-Cys^−^Na^+^ and (**b**) SG-Cys^−^Ag^+^.

Scanning Electron Microscopy (SEM) offers valuable topographical insights, allowing us to discern surface characteristics of materials such as SG-Cys^−^Na^+^ and SG-Cys^−^Ag^+^. Initially, the SG-Cys^−^Na^+^ surface exhibited a pristine, smooth appearance (see Figure 3a). However, the introduction of silver ions onto SG-Cys^−^Na^+^ led to noticeable alterations in surface morphology. Most notably, the emergence of particles or clusters, measuring approximately 19.4 nm in size, was observed. A comparison of the sizes of these white features on both SG-Cys^−^Na^+^ and SG-Cys^−^Ag^+^ surfaces indicates a shift in elemental composition, from sodium to silver ions. Furthermore, these changes brought about modifications in texture, appearance, and color (refer to Figure 3b). These observations indicate the capture of silver ions and the formation of SG-Cys^−^Ag^+^.

### 3.4. XRD

**Figure 4:**
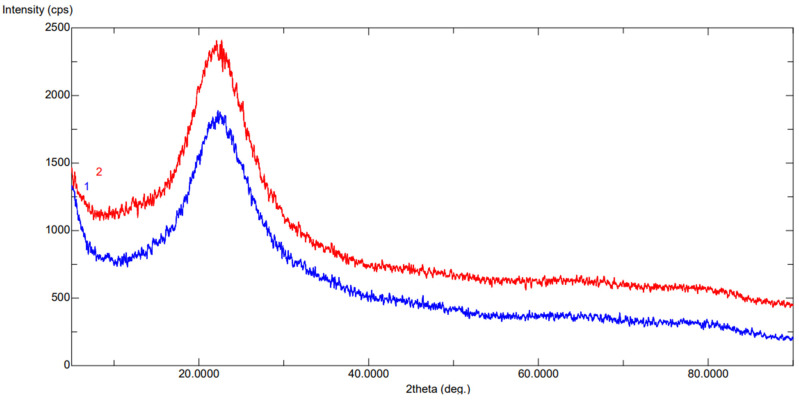
The XRD of (2) SG-Cys^−^Na^+^ and (1) SG-Cys^−^Ag^+^.

X-ray diffraction (XRD) is commonly used to analyze silica materials due to its ability to provide valuable information about their crystal structure, phase composition, and lattice parameters. Figure 4 shows the XRD spectrum for both SG-Cys^−^Na^+^ and SG-Cys^−^Ag^+^ materials. Both of them have a broad peak at 2θ = 22.4°. The spectrum shows no change in the phase composition and the degree of crystallinity of the materials. The absence of peaks related to the Ag_2_O and Ag(0) provides evidence that the silver is doped inside the matrix as a silver (I) ion [34].

### 3.5. Effect of pH

**Figure 5:**
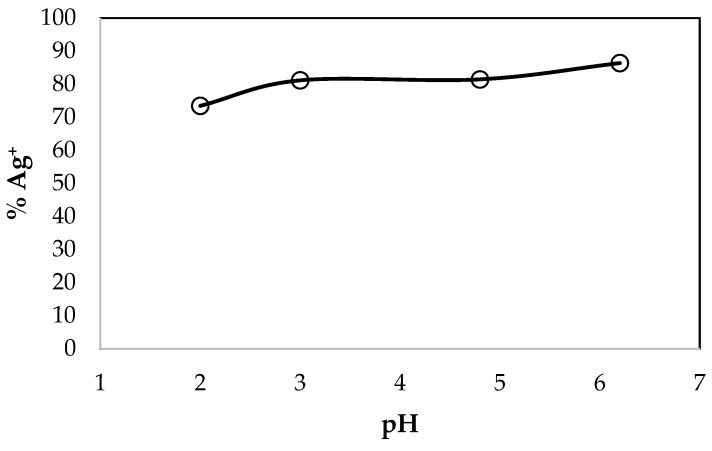
Effect of initial pH on the capturing of silver ion Ag^+^ onto SG-Cys^−^Na^+^ (*C_i_* = 50 mg L^−1^, *T* = 25 °C, 80 rpm, dosage = 2 g L^−1^, *t* = 180 min).

The pH of the solution can influence the capture of Ag(I) ions in different ways. The concentrations of hydrogen ions (H^+^) and hydroxide ions (OH^−^) in a solution are affected by the pH, which affects the surface charge of the SG-Cys^−^Na^+^ and the speciation of the silver ions. Figure 5 depicts the influence of solution pH on the loading of silver ions into the SG-Cys^−^Na^+^ matrix with increasing pH and loading capacity. The presence and concentration of other ions that may compete with silver ions for capture sites can also be affected by the pH of the solution. For example, at low pH, hydrogen ions (H^+^) are abundant and may compete with silver ions for binding sites on the adsorbent [35]. The highest loading percentage on the surface of SG-Cys^−^Na^+^ was 86% at pH 6. At higher pH, silver hydroxide is formed. Wherein, silver hydroxide is sparingly soluble in water, and its solubility is relatively low. This leads to the precipitation of silver ions, which affects the availability of silver ions for loading into SG-Cys^−^Na^+^.

### 3.6. Effect of Initial Metal Ion Concentration

**Figure 6:**
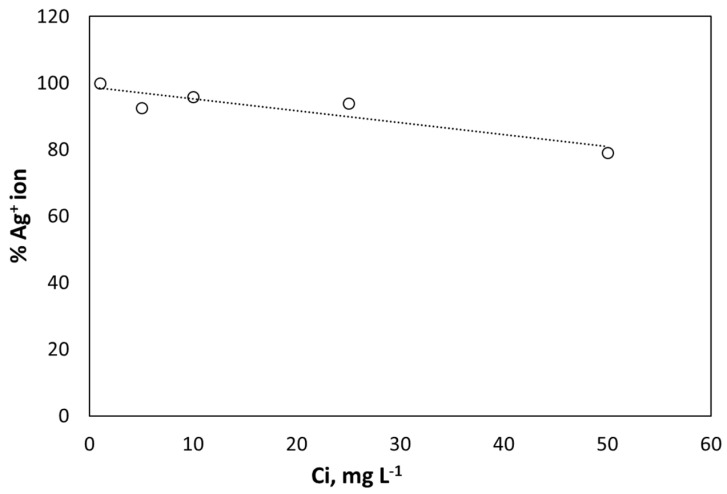
Effect of initial concentration on the capturing of silver ion Ag(I) onto SG-Cys^−^Na^+^ (*T* = 25 °C, 80 rpm, dosage = 2 g L^−1^, *p*H_i_ = 6.0, *t* = 40 min).

The initial Ag(I) concentration can have a substantial impact on the SG-Cys^−^Na^+^ capture process. The concentration of Ag(I) ions in the initial solution affected the full loading capacity, efficiency, and kinetics of the process. The initial concentration of Ag(I) is shown in Figure 6. Lower starting concentrations resulted in more potential capturing sites on the adsorbent surface, resulting in larger capturing capacities. The number of potential sites may become limited as the initial concentration increases, resulting in a decrease in catching capacity. Furthermore, the initial concentration of silver ions can affect the SG-Cys^−^Na^+^ capture efficiency, which refers to the proportion of Ag(I) ions efficiently extracted from the solution. Higher initial concentrations may result in a higher number of Ag(I) ions being adsorbed initially because of the larger driving force for capture. However, as the initial concentration increases, the ion exchange capture efficiency may decrease owing to the increased competition between Ag(I) ions for the available SG-Cys^−^Na^+^ sites, as has been reported [36]. In addition, at higher metal ion concentrations, the electrostatic repulsion between Ag(I) ions can also reduce the capturing efficiency of SG-Cys^−^Na^+^. The electrostatic repulsion between similarly charged silver ions can hinder their capture on the SG-Cys^−^Na^+^ surface, which ultimately decreases the overall capturing efficiency.

### 3.7. Effect of Temperature

**Figure 7:**
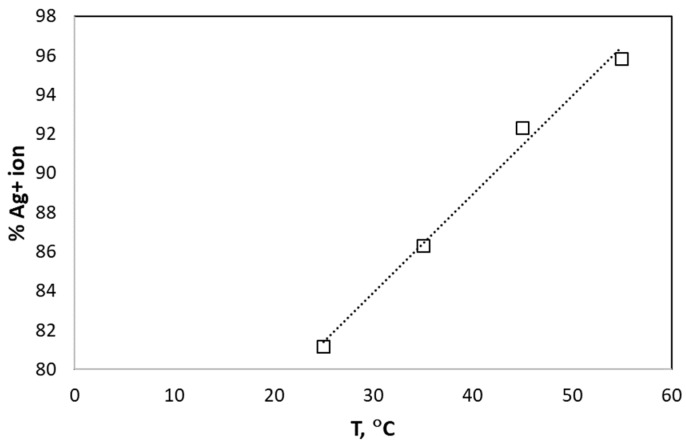
Effect of Temperature on the capturing of silver ion Ag(I) onto SG-Cys^−^Na^+^ (*C_i_* = 50 mg L^−1^, *t* = 180 min, 80 rpm, dosage = 2 g L^−1^, *p*H_i_ = 6.0).

Temperature variations can also affect the capture mechanism. Different temperature ranges may promote different capture mechanisms, such as physical or chemical bonding. The interaction between Ag(I) ions and the functional groups present on the SG-Cys^−^Na^+^ surface can be temperature-dependent, leading to variations in the capturing behavior. Therefore, the temperature can significantly influence the capture of Ag(I) onto SG-Cys^−^Na^+^. Temperature changes affect the capturing capacity, kinetics, and overall capturing process. Generally, increasing the temperature leads to an increase in the ion exchange capacity of Ag(I) onto SG-Cys^−^Na^+^ (see Figure 7), which is consistent with the reported results [37]. This is because higher temperatures provide more thermal energy, which enhances the diffusion of Ag(I) ions from the solution onto the SG-Cys^−^Na^+^ surface. The optimal temperature for maximizing the capture efficiency and capacity of Ag(I) onto SG-Cys^−^Na^+^ was T = 55 °C (96%). The chemical interaction between silver ions and silica gel is typically exothermic. Increasing the system’s temperature provides additional energy to the system, which can increase the reaction rate and improve the capture efficiency.

### 3.8. Effect of Contact Time vs. pH

The contact time between SG-Cys^−^Na^+^ and Ag(I) ions can have a significant effect on the capturing efficiency of the process. The optimal contact time for capturing Ag(I) ions by SG-Cys^−^Na^+^ was determined experimentally by measuring the sorption capacity of SG-Cys^−^Na^+^ at different time intervals. Generally, as the contact time increased, the amount of Ag(I) ions adsorbed onto SG-Cys^−^Na^+^ also increased until equilibrium was reached. At the beginning of the capture process, the matrix network of SG-Cys^−^Na^+^ was relatively unoccupied by Ag(I) ions. Therefore, there are many available sites for chemisorption, and the initial capture rate is very fast. As the matrix becomes more occupied, the number of available sites for capturing decreases, and the capturing rate slows until equilibrium is reached. The equilibrium point is reached when the rate of capture is equal to the rate of desorption, and no further net change in the amount of Ag(I) ions adsorbed onto SG-Cys^−^Na^+^ occurs. This equilibrium point is affected by various factors, such as the concentration of Ag(I) ions in the solution, pH, temperature, and properties of the silica gel. 

**Figure 8:**
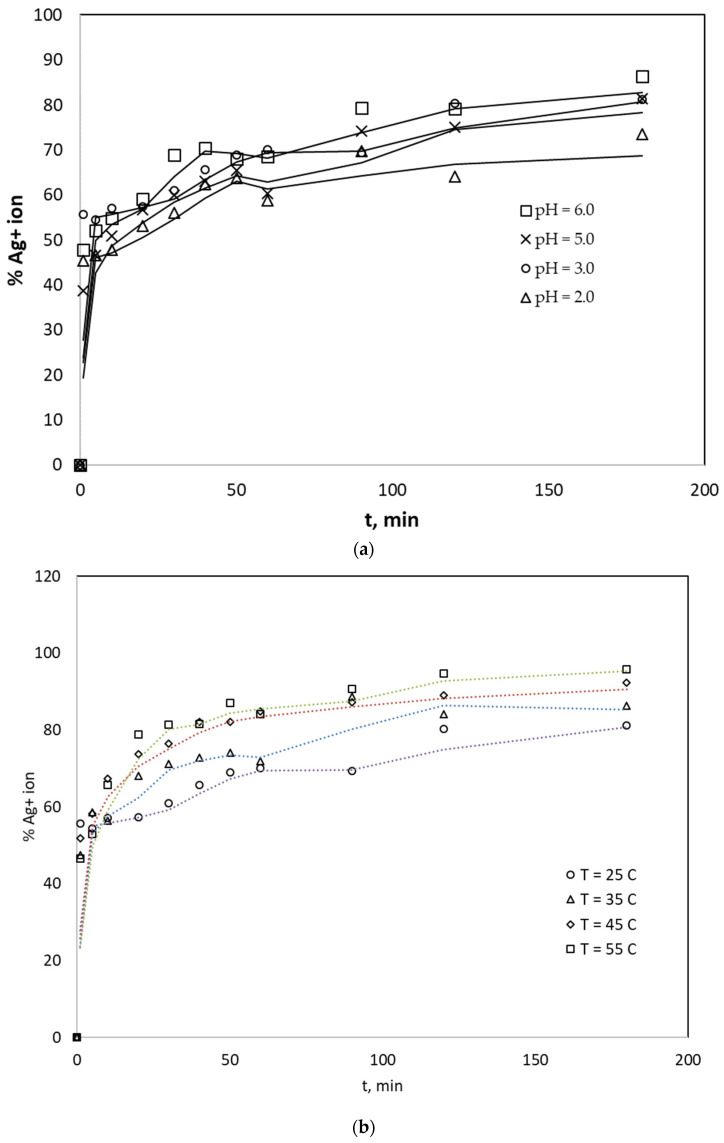

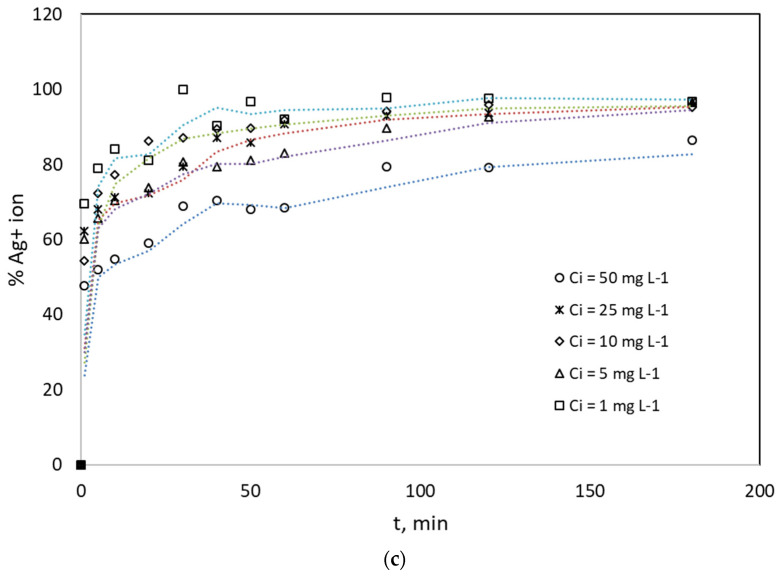
(**a**) Effect of contact time on the capturing of silver ion Ag(I) onto SG-Cys^−^Na^+^ (*C_i_* = 50 mg L^−1^, *T* = 25 °C, 80 rpm, dosage = 2 g L^−1^). (**b**) Effect of contact time on the capturing of silver ion Ag(I) onto SG-Cys^−^Na^+^ (*C_i_* = 50 mg L^−1^, *p*H_i_ *= 6,* 80 rpm, dosage = 2 g L^−1^). (**c**) Effect of contact time on the capturing of silver ion Ag(I) onto SG-Cys^−^Na^+^ (*p*H_i_ = 6, *T* = 25 °C, 80 rpm, dosage = 2 g L^−1^).

Figure 8a,b. showed that the optimal contact time for capturing Ag(I) ions by SG-Cys^−^Na^+^ can be determined experimentally up to 180 min, achieving 86% at *p*H_i_ = 6.0 without reaching equilibrium (Figure 8a). The optimal contact time for equilibrium was 50 min, achieving 98% capture of Ag(I) ions at a low initial concentration of 1 mg L^−1^, as shown in Figure 8c, even at various temperatures (Figure 8b). In general, increasing the temperature of a system can enhance the kinetic energy of the molecules, which leads to an increased frequency of collisions between silver (I) ions and the SG-Cys^−^Na^+^ surface. This can result in a higher probability of silver (I) ions being adsorbed onto the surface of the SG-Cys^−^Na^+^, increasing the capturing efficiency. Moreover, higher temperatures typically accelerate capture kinetics, as they provide more energy for the capturing process. The increased thermal energy increases the Ag(I) ion mobility, allowing it to diffuse more quickly onto the surface of SG-Cys^−^Na^+^. As a result, at higher temperatures, the time required to reach capture equilibrium is shortened. This finding matches previously reported [32,38].

From a kinetic standpoint, the observed behavior can be attributed to the influence of initial silver ion concentration on the contact time required to reach equilibrium. At a low initial concentration of 1 mg L^−1^, the surface reaction between SG-Cys^−^Na^+^ and Ag(I) ions reaches equilibrium relatively quickly, with an optimal contact time of 50 min, resulting in a high capture efficiency of 98%. In contrast, when dealing with higher initial concentrations (50 mg L^−1^), it takes significantly longer (>180 min) to attain equilibrium, and the capture efficiency is lower, reaching only 86%. This delay in equilibrium attainment at higher concentrations can be attributed to the increased number of silver ions competing for binding sites on the sorbent material, leading to a slower rate of sorption until saturation is achieved [36,37].

### 3.9. Sorption Kinetics

Pseudo-first-order and pseudo-second-order models are used to describe the sorption kinetics of metal ions onto solid sorbents, including silica gel, in the context of the sorption of silver (I) ions onto SG-Cys^−^Na^+^. These models could be used to describe the sorption rate. The pseudo-first-order rate equation is as follows [39].

(1) $$\ln(q_{e}-q_{t})=\ln q_{e}-k_{1}t\;$$

The pseudo-first-order model assumes that the rate of capture decreases as the number of unoccupied surface sites on silica gel decreases, leading to a decrease in the rate of silver ion uptake over time. The model is useful in describing the initial stages of sorption when the concentration of silver ions in solution is high and the number of capturing sites on SG-Cys^−^Na^+^ is not saturated. However, when the calculated R^2^ < 0.165, it may not accurately describe the sorption process. Therefore, another model can be used as a pseudo-second-order model. The pseudo-second-order model assumes that the rate-limiting step for the sorption process is the chemical reaction between the silver ions and functional groups on the SG-Cys^−^Na^+^ surface. This model is useful in describing the entire process of sorption, from the initial stages when the concentration of silver ions in the solution is high to the later stages when the surface area of the SG-Cys^−^Na^+^ becomes fully occupied by silver ions. This can be expressed mathematically as [38]:

(2) $$\frac{t}{q_{t}}=\frac{1}{k_{2}q_{e}^{2}}+\frac{t}{q_{e}}\;$$

where in, *t* (minutes) is contact time. Both *q_e_* and *q_t_* (mg g^1^) are the amounts of adsorbed Ag(I) ions at equilibrium and at the time (*t*), respectively. *k*_1_ (min^−1^) is the pseudo-first-order rate constant, *k*_2_ is the rate constant of the pseudo-second-order kinetic model (g mg^−1^ min^−1^). From the experimental data, a plot *t*/*q*_t_ versus *t* was used to obtain a linear plot. The slope of the plot is equal to 1/*q_e_*, and the intercept is equal to 1/(*k*_2_ × *q_e_*^2^). 

**Figure 9:**
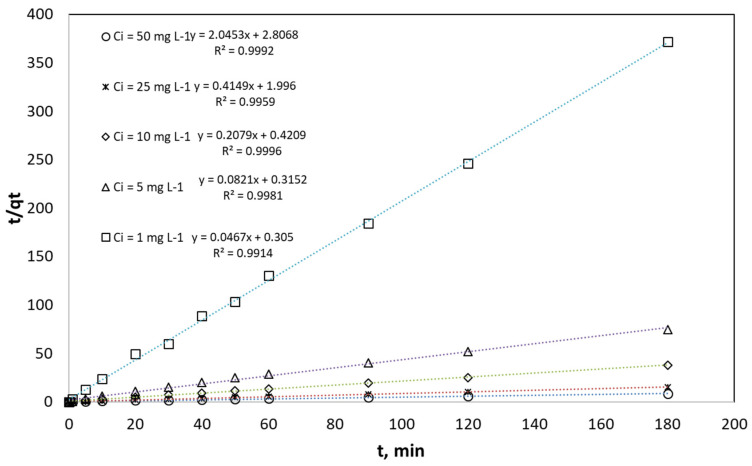
The pseudo-second-order model for the sorption of Ag(I) onto SG-Cys^−^Na^+^ different *C_i_* (*p*H_i_ *= 6*, *T* = 25 °C, 80 rpm, dosage = 2 g L^−1^).

The rate constant for the sorption process was estimated by calculating *k*_2_ from the intercept of the plot. The value of *k*_2_ can be used to predict the number of silver ions sorbed at any time t as well as the equilibrium sorption capacity *q_e_*. To apply this model to the sorption of silver ions onto SG-Cys^−^Na^+^, batch sorption experiments at different initial silver ion concentrations and temperatures were conducted. The number of silver ions sorbed at various time intervals was measured. Figure 9 shows that a pseudo-second-order model is preferred over the pseudo-first-order model because it provides a better fit to the experimental data over a wider range of temperatures or initial concentrations and time intervals. The pseudo-second-order model (R^2^ > 0.999) assumed that the capture rate was controlled by chemisorption. This involves the formation of a chemical bond between the silver ion and the surface of SG-Cys^−^Na^+^, as confirmed by FTIR.

**Figure 10:**
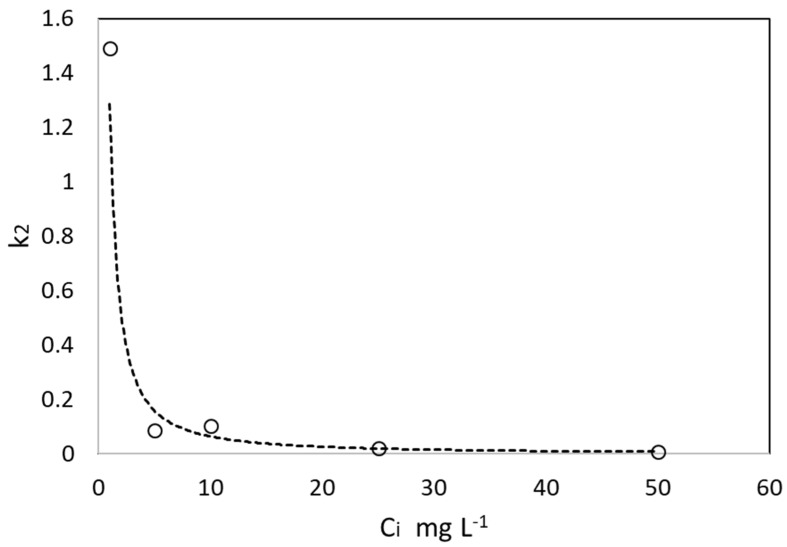
The rate constant of the pseudo-second-order kinetic model (g mg^−1^ min^−1^) for the sorption of Ag(I) onto SG-Cys^−^Na^+^ different *C_i_*.

In a pseudo-second-order kinetic model, the rate constant (*k*_2_) describes the rate at which a reaction occurs. Specifically, it represents the rate of the reaction between a silver ion and SG-Cys^−^Na^+^ functional groups, which typically follows second-order reaction kinetics. When the initial concentration of silver ions was increased, the rate constant of the pseudo-second-order kinetic model tended to decrease, as shown in Table 1 and Figure 10. This behavior can be attributed to the fact that as the initial concentration of the silver ion increases, the reaction becomes more dependent on the availability of SG-Cys^−^Na^+^, rather than the silver ion itself. Therefore, at higher silver ion concentrations, SG-Cys^−^Na^+^, became more saturated or depleted, leading to a decrease in the reaction rate constant.

**Figure 11:**
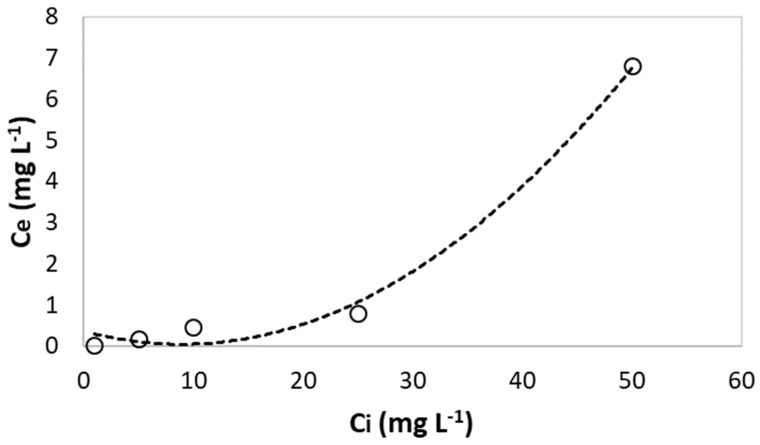
The plot of *C_i_* (initial concentration) versus *C_e_* (concentration at equilibrium) in a sorption process typically shows the change in concentration of a silver ion or SG-Cys^−^Na^+^ over time.

The plot of *C_i_* (initial concentration) versus *C_e_* (concentration at a specific time) in a sorption process typically shows the change in concentration of a silver ion or SG-Cys^−^Na^+^ over time as shown in Figure 11. This type of plot helps to visualize the behavior of the sorption process and provides insight into the capturing efficiency of the SG-Cys^−^Na^+^. In general, the plot may exhibit a downward trend as the concentration of the silver ion decreases over time due to its capture onto the SG-Cys^−^Na^+^. The rate at which the concentration of silver ions decreases can provide information about the kinetics of the sorption process, such as whether it follows a first-order or second-order reaction. plotting *C_i_* versus *C_e_* in a sorption process helps analyze the concentration changes and understand the dynamics of the SG-Cys^−^Ag^+^ interaction over time.

### 3.10. Capturing and Sorption Isotherm

**Figure 12:**
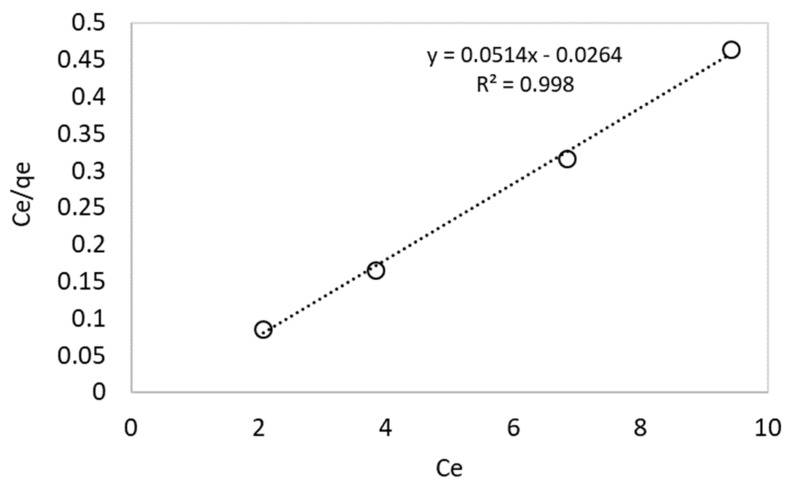
Langmuir isotherm for the sorption of Ag(I) onto SG-Cys^−^Na^+^ different *T*.

**Figure 13:**
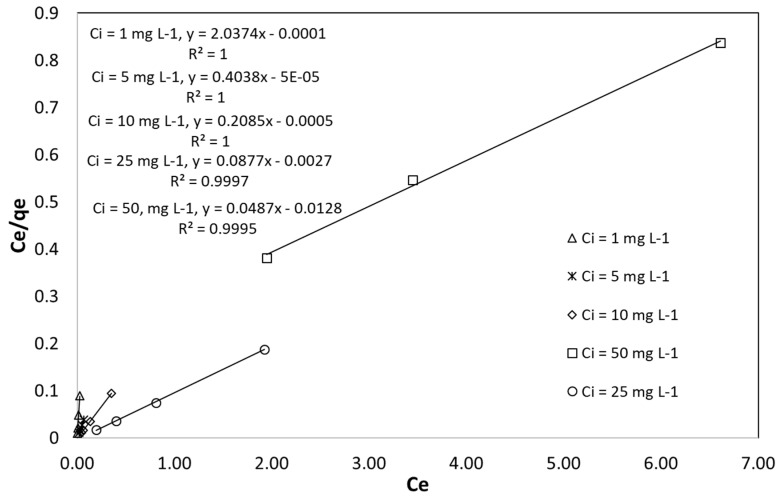
Langmuir isotherm for the sorption of Ag(I) onto SG-Cys^−^Na^+^ different *C_i_*.

The Langmuir isotherm is a theoretical model that depicts the sorption of an Ag(I) ion onto a surface composed of SG-Cys^−^Na^+^. As shown in Figure 12 and Figure 13, it is assumed that the sorption occurs on a homogenous SG-Cys^−^Na^+^ surface and that each sorption site can only accommodate one Ag(I) ion. The Langmuir isotherm equation is as follows [40]:

(3) $$\frac{C_{e}}{q_{e}}=\frac{1}{q_{max}b}+\frac{1}{q_{max}}C_{e}\;$$

and, the Freundlich isotherm is an empirical equation that describes the sorption of an Ag(I) onto a solid phase, such as SG-Cys^−^Na^+^. It is commonly used to model the capturing of ions onto porous materials, including silver ions onto SG-Cys^−^Na^+^. The Freundlich isotherm equation can be expressed as [41]:

(4) $$\ln q_{e}=\ln K_{F}+\left(\frac{1}{n}\right)\ln C_{e}$$

where $q_{e}\;$is the amount of Ag(I) ion adsorbed per unit mass of SG-Cys^−^Na^+^ (usually at the equilibrium and expressed in mg g^−1^), *C_e_* is the equilibrium concentration of the Ag(I) in the solution (usually expressed in mg L^−1^), $q_{max}\;$is the maximum amount of solute that can be adsorbed per unit mass of adsorbent (also known as the Langmuir capacity, expressed in mg g^−1^), and *b* is the Langmuir constant (expressed in L mg^−1^).$\;K_{f}$ (mg g^−1^), and *n* are the Freundlich constants that describe the sorption capacity and intensity of the SG-Cys^−^Na^+^, respectively.

To determine the Freundlich constants for the sorption of silver ions onto SG-Cys^-^Na^+^, experimental data were collected by equilibrating a known amount of SG-Cys^−^Na^+^ with varying concentrations of silver ion solution. The amount of silver ion adsorbed onto the SG-Cys^−^Na^+^ was measured, and the data can be fitted to the Freundlich isotherm equation to determine the values of K and n. These constants can be used to predict the number of silver ions adsorbed onto the SG-Cys^−^Na^+^ at different concentrations of silver ion solution.

In the Langmuir isotherm of sorption, the parameter “b” represents the equilibrium constant or the binding constant. This parameter is indicative of the strength of adsorption between the silver ions and the SG-Cys^−^Na^+^. A higher value of “b” indicates a stronger affinity between the silver ions and the SG-Cys^−^Na^+^. Table 2 shows a negative Langmuir constant (*b*), which may occur when the capturing process is exothermic. This means that the heat released during capturing is greater than the heat required for desorption. In this case, the capturing equilibrium constant decreases with increasing temperature, resulting in a negative Langmuir constant. It is important to note that a negative Langmuir constant is relatively rare and is not commonly observed in sorption processes.

### 3.11. Thermodynamic Parameters

Since the reaction mechanism follows the Langmuir isotherm, the standard Gibbs free energy change (Δ*G*°) for the captured silver ion can be calculated by Equation (5):

(5) $$\Delta G=-RTlnK_{l}$$

where in, *R* is the universal gas constant (8.314 J mol^−1^ K^−1^), *T* is the absolute temperature in Kelvin and *b* is the equilibrium constant, related to the Langmuir constant (*b*) as listed in Table 2. The thermal parameters Δ*H* and Δ*S* can be calculated by using Vant Hoff linear Equation (6) [42]:

(6) ΔG=ΔH−TΔS

The initial solute concentration can affect the thermodynamic parameters of the sorption. Thermodynamic parameters describe the energetics and equilibrium behavior of the sorption process. Table 3 shows that higher initial concentrations can lead to higher equilibrium constants, indicating a stronger affinity between the solute and sorbent. Higher initial concentrations often resulted in more negative Δ*G* values, indicating a more favorable and spontaneous sorption process. Higher initial concentrations can lead to more positive Δ*S* values, indicating an increase in system disorder.

Finally, it is necessary to comment on the results of the recent study that was published recently [43], it has been reported the synthesis of thiol-functionalized porous silica particles employing (3-mercaptopropyl)-trimethoxy-silane (MPTMS), resulting in the formation of SG-(3-mercaptopropyl)-trimethoxy-silane (SG-MPTMS) [43]. This material differs significantly from utilizing L-Cysteine-Functionalized Silica Gel Material (SG-Cys^−^Na^+^). While both materials have been utilized for silver ion capture, the recent study (SG-MPTMS) establishes that silver ions are primarily bound to the thiol functional group. In contrast, our investigation suggests that silver ions are predominantly bound to SG-Cys^−^Na^+^ via the carboxyl group situated on the cysteine molecule. It is noteworthy that both SG-MPTMS and SG-Cys^−^Na^+^ incorporate thiol functional groups, but the mechanism for silver ion binding diverges between them. Thiol groups appear to facilitate silver ion capture through SG-MPTMS, whereas silver ions may interact with carboxylate active sites in the case of our SG-Cys^−^Na^+^. By addressing these distinct research findings and methodological disparities, our collective efforts contribute significantly to advancing our respective research domains and offer practical solutions to real-world challenges.

## 4. Conclusions

In conclusion, the research study has demonstrated the successful loading of silver (Ag(I)) ions onto a specially functionalized silica matrix, known as SG-Cys^−^Na^+^, using the batch sorption technique. The optimized conditions for achieving maximum Ag(I) loading efficiency were found to be at a *p*H_i_ of 6, a stirring speed of 80 rpm, an initial silver ion concentration of 1 mg L^−1^, and a temperature of 55 °C, resulting in an impressive 98% loading efficiency. The Langmuir isotherm model is employed to describe the adsorption behavior, and it was found to be highly suitable, showing a good fit with the experimental data (*R*^2^ = 0.999). This suggests the formation of a homogeneous monolayer of silver ions on the SG-Cys^−^Na^+^ active sites, as confirmed by FTIR spectroscopy. Additionally, XRD analysis indicated the stability of the silica matrix and the absence of undesirable Ag_2_O and Ag(0) phases. The kinetic study revealed that the loading process followed a pseudo-second-order model (*R*^2^ > 0.999), implying that chemisorption played a crucial role in binding silver ions to the SG-Cys^−^Na^+^ surface. This chemisorption involves strong chemical bonding between the silver ions and the carboxylate groups (-COO^-^Na^+^) into functionalized silica gel. Furthermore, the thermodynamic parameters calculated in this study indicated that higher initial silver ion concentrations led to increased equilibrium constants, negative Gibbs free energy (ΔG) values, positive entropy change (ΔS) values, and negative enthalpy change (ΔH) values. These findings suggest that the loading of silver(I) ions onto the SG-Cys^−^Na^+^ matrix is a spontaneous and exothermic process.

This study not only provides valuable insights into the potential application of the SG-Cys^−^Na+ matrix as an efficient filter for silver ion removal from aqueous solutions but also contributes to the advancement of sustainable and environmentally friendly water treatment technologies. Furthermore, the immobilization of silver ions within the SG-Cys^−^Na+ matrix forms a composite material with potential uses in catalytic and conductive applications.

## Figures and Tables

**Table 1:** The characteristic parameters of the pseudo-second-order model.

*C_i_* (mg L^−1^)	*k*_2_ (L mol^−1^ s^−1^)	*q*_e cal._	*q*_max exp._	*R*²
1	1.490399	0.488926	0.484173	0.9992
5	0.086243	2.410219	2.411511	0.9959
10	0.10269	4.810005	4.761871	0.9996
25	0.021385	12.18027	12.10072	0.9981
50	0.00715	21.41328	21.59618	0.9914

**Table 2:** Langmuir isotherm constant in various initial concentrations.

*C_i_,* mg L^−1^	*q*_cal. max_	*b, L* mg^−1^	*R*²
**1**	0.490822	−20,374	1
**5**	2.476474	−8076	1
**10**	4.796163	−417	1
**25**	11.40251	−32.4815	0.9997
**50**	20.53388	−3.80469	0.9995

**Table 3:** Thermodynamic parameters for the sorption of Ag(I) into SG-Cys^−^Na^+^ at different *C_i_.*

*C_i_*, mg L^−1^	Δ*H,* kJ mol^−1^	Δ*S,* J mol^−1^ K^−1^	T, *K*	Δ*G*, J mol^−1^
**5**	−35,051	+149.9347	298.15	−79,754
**10**	−58,227.9	+221.6762	298.15	−124,321
**25**	−63,656.1	+233.4322	298.15	−133,254

## Data Availability

All data generated or analyzed during this study are included in this published article.
